# Treatment of Retained Fetal Membranes in the Mare—A Practitioner Survey

**DOI:** 10.3389/fvets.2018.00128

**Published:** 2018-06-19

**Authors:** Dinuka N. Warnakulasooriya, Christina D. Marth, Jacqueline A. McLeod, David W. Hanlon, Natali Krekeler

**Affiliations:** ^1^Asia-Pacific Centre for Animal Health, Faculty of Veterinary and Agricultural Sciences, The University of Melbourne Werribee, VIC, Australia; ^2^Matamata Veterinary Services Ltd. Matamata, New Zealand

**Keywords:** retained fetal membranes, horse diseases, equine, post-partum disease, retained placenta

## Abstract

Retained fetal membranes (RFM) is a common post-partum problem in mares for which the treatment is highly variable. The aim of this study was (i) to investigate the different treatments used by equine practitioners for RFM and (ii) to determine if there is a difference between treatments used by reproductive specialists and general equine practitioners. Information regarding treatment of RFM was sought from veterinary practitioners via a survey and this was compared to recommendations in the current literature. The survey was sent out to equine veterinarians and mixed practitioners with a high equine case load. Most treatments of RFM were in line with current recommendations, while some obsolete practices are still routinely performed by a small number of practitioners. Treatment recommendations for RFM have changed over the last few decades, but there are no universally accepted guidelines. The vast variety of treatments reported by practitioners in the present survey reflect this lack of guidance. More extensive research is needed in this area to establish evidence-based, uniformly agreed upon protocols.

## Introduction

Retained fetal membranes (RFM) is the most common post-partum problem in mares (1). RFM is defined as the complete or partial failure to release the chorioallantois within a pre-defined timeframe post-partum. The duration of time before the membranes are considered to be retained varies widely from 30 min to up to 24 h (2–4). A recent study found that 95% of Thoroughbred mares expelled their membranes within 4 h, whereby more than two-thirds of the mares had already expelled them after 1 h (5).

The cause of RFM is not known for certain, but a combination of uterine inertia and hormonal imbalances has been suggested previously (4). Other causes that have been reported include serum calcium and phosphate imbalances, dysregulation of extracellular matrix remodeling and activation, physical intervention, placental infection, and/or edema, trauma to endometrial tissue and uterine infections (4, 6–8). Pre-disposing factors for uterine inertia include low blood calcium levels, overstretching of the myometrium due to twins, myometrial degeneration due to infections, and myometrial exhaustion in the course of dystocia. Hormonal imbalances have been attributed to low oxytocin receptor expression (9).

There are many factors that have been reported to pre-dispose mares to develop RFM. These include draft horses including Friesians (6), mares older than 15 years and those with a history of RFM (10). Inbreeding has also been shown to play a role in the high incidence observed in Friesian mares (11). Additionally, uterine or systemic infections before or during pregnancy, poor body condition, additional reproductive problems such as dystocia or prolonged gestation and artificial intervention, particularly cesarean sections contribute to an increased risk for RFM (7, 12). Some of these pre-disposing factors such as age and gestation length have been disputed (6). The most prominent pre-disposing factors to RFM seem to be Friesian breed and a previous history of RFM (6, 10). A study conducted by Sevinga et al. (6) found that Friesians had a 54% incidence rate of RFM compared to an average of 2–10% in non-draft breeds. The same study found that mares with a previous episode of RFM had a 2.9-fold increased chance of developing the condition again (6). Fertility parameters in subsequent pregnancies do not differ between mares suffering from RFM compared to normal mares (6).

Complete or partial (commonly of the non-fetal horn) fetal membrane retentions are usually diagnosed by visual observation of fetal membranes protruding from the vagina, vaginal palpation, and/or clinical signs such as colic. Further diagnostic tests may be indicated to evaluate the state of the patient, particularly regarding a developing septicemia (4). Potential complications include toxic metritis, septicemia, endotoxemia, laminitis, and possibly death (12–14). Timely treatment is essential to avoid these complications.

Treatment suggestions are the most debated aspect of RFM both in the literature and amongst practitioners. The main aims of treatments are to eliminate toxic and inflammatory products from the uterus, control systemic shock symptoms and endotoxemia, and prevent laminitis (7).

Oxytocin, antibiotics, and uterine lavage are considered the most essential treatments with oxytocin being the most commonly reported initial treatment (14). Oxytocin is an uterotonic hormone that encourages uterine contractions leading to the expulsion of RFM. However, in draft breeds and under certain aggravating conditions, it should be used cautiously (4). The recommended routes, dosage and length of treatment vary widely. Oxytocin can be administered as a slow intravenous (IV) drip, bolus IV injection, and intramuscular (IM) injection (4). The recommended dose ranges from 2 to 120 IU. Doses >20 IU as a bolus are not recommended due to the potential to cause severe abdominal pain and tonic uterine spasms (7, 12). Oxytocin is usually given for no more than a 24 h period (4). The addition of calcium borogluconate alongside oxytocin has been suggested due to an improved response observed in mares and the detection of low serum ionized calcium levels in mares with RFM (6). The administration of broad spectrum antibiotics to all mares experiencing RFM is often recommended to prevent bacterial growth *in utero* and secondary septicemia/endotoxemia (1, 3, 4, 6, 7, 12, 14). A combination of beta-lactam antibiotics (e.g., penicillin) and aminoglycosides (e.g., gentamicin) covers most of the common pathogens found in the equine uterus (7). The duration of the antimicrobial therapy varies but should continue for 1–2 weeks post-RFM expulsion (4, 7). Some authors recommend the concurrent use of systemic and intrauterine antibiotics. Intrauterine administration of oxytetracycline starting 8 h post-foaling is one such option (4). It has to be noted that the use of oxytetracycline in non-pregnant mares was associated with endometritis (15). Uterine lavage can be carried out prior to or after RFM expulsion. Small pieces of membranes remaining in the uterus can be flushed out by uterine lavage (16, 17). It is thought to help to physically remove bacteria and cell debris, stimulate uterine contractility and attract neutrophils to the uterus (7). Several lavage fluids and techniques have been described. One well-known technique is the “Burns Technique” involving the introduction of large volumes of lavage fluid into the allantoic cavity followed by the manual occlusion of its entrance for 10–15 min (18). Lavage is usually repeated until the returning fluid is clear. Some authors recommend the use of antiseptics in the lavage fluid, but this may cause severe uterine irritation (4). Ringer's solution, isotonic saline, and tap water are all routinely used for uterine lavage (4, 19, 20).

Additional treatments include non-steroidal anti-inflammatory drugs (NSAIDs) used against inflammation, endotoxemia, and pain (14). Tetanus toxoids or anti-toxins and IV fluid therapy are also highly recommended (4, 7). Laminitis prevention includes ice boots, NSAIDs, and pentoxifylline administration (7).

The most controversial treatment is manual removal of the membranes. To date there is no convincing scientific evidence for its benefits. Several publications recommend manual removal of the membranes suggesting a quick resolution of the condition (17, 21). Other authors oppose this practice due to the increased chance of absorption of septic/toxic materials and delayed uterine involution (4, 12). Possible complications include hemorrhage, uterine horn invagination, delayed uterine involution, pulmonary emboli, and permanent endometrial damage (4). One of the biggest concerns amongst practitioners is fertility post-RFM removal. One study comparing fertility parameters of mares which underwent manual removal of membranes to those that expulsed the fetal membranes spontaneously reported no statistical difference between the groups (17). However, mares underwent membrane removal immediately post-parturition and can therefore not be compared with mares suffering from RFM. Samper et al. (22) reported that ambulatory veterinarians routinely remove RFM manually because less revisits are required. They suggest this as an acceptable method as long as it is being done carefully (22).

With appropriate and timely treatment, the prognosis for survival and future fertility for mares with RFM is excellent. Mares that develop metritis, endotoxemia, and/or laminitis have poor to moderate long term survival (7). This shows the necessity of guidelines to enable veterinarians to treat all mares with RFM using evidence-based techniques published in the literature. To determine the current status of treatments, a survey was conducted to (i) evaluate which treatments were used for RFM by equine practitioners and (ii) to determine if reproductive specialists and general equine practitioners treat mares with RFM differently. These results can be used to inform recommendations and guidelines in the future.

## Materials and methods

### Study population

The target study population consisted of veterinarians self-reporting spending a significant proportion of their time treating horses. Both veterinarians in mixed practice with an equine emphasis and equine only practices were included in the study. Veterinarians in this study had a diverse set of qualifications ranging from general practitioners to reproductive specialists and are practicing in several different countries. These factors were adjusted for in our analysis.

### Questionnaire

Seventeen questions captured the respondents' backgrounds, as well as the techniques they used for the diagnosis and treatment of RFM, complications, and pre-dispositions they associated with RFM. Most of the questions were open-ended with an unlimited word count comment box provided (see Supplementary Material).

### Survey delivery

The survey was distributed in a web-based online format using the eSurveyCreator® platform and as attachment to emails in Microsoft Word®. The survey was conducted over 2 years by two separate authors. The first author conducted the survey over a 6 week period in 2013 while the second author conducted the same survey again in 2014 over a 5 week period. Respondents were chosen via specialist and non-specialist Listservs, personal contacts, the Australian Veterinary Association, the American Association of Equine Practitioners, and websites of several veterinary clinics.

### Analysis

The four dependent variables of interest were (a) the number of hours post-foaling when fetal membranes were considered to be retained, (b) the proportion of practitioners that attempted manual removal of fetal membranes, (c) the proportion of practitioners using the “Burns technique,” and (d) the dose of oxytocin used to treat RFMs.

The independent variables included in each multivariable model were: country of practice for each practitioner, years in equine practice, and whether the practitioner was a specialist. A generalized linear model (GLM) was used to examine the effects of the independent variables on the binomial outcome variables of the proportion attempting manual removal and the proportion using the Burns technique. All independent variables (including interactions) were initially included (full model) and then a backward, step-wise model building process was used where non-significant (Wald's-test, *P* > 0.05) interactions were removed sequentially, followed by non-significant main effects. After each variable was dropped from the model, a likelihood ratio test was performed to compare the new, reduced model to the previous model, to determine significance of the reduced model. Multivariable linear regression was used to examine the effects of the independent variables on the linear outcome variables of hours to retained membranes and oxytocin dose, with model-building proceeding as for the GLMs. All analyses were performed using R version 3.3.2 (R development core team, 2016; R foundation for statistical computing, Vienna, Austria, http://www.r-project.org). Significance was determined at *P* < 0.05.

### Ethics

The survey and questionnaires were reviewed by the Veterinary Science Human Ethics Advisory Group and approved as a minimal risk project (Ethics ID: 1339340).

## Results

**Figure 1:**
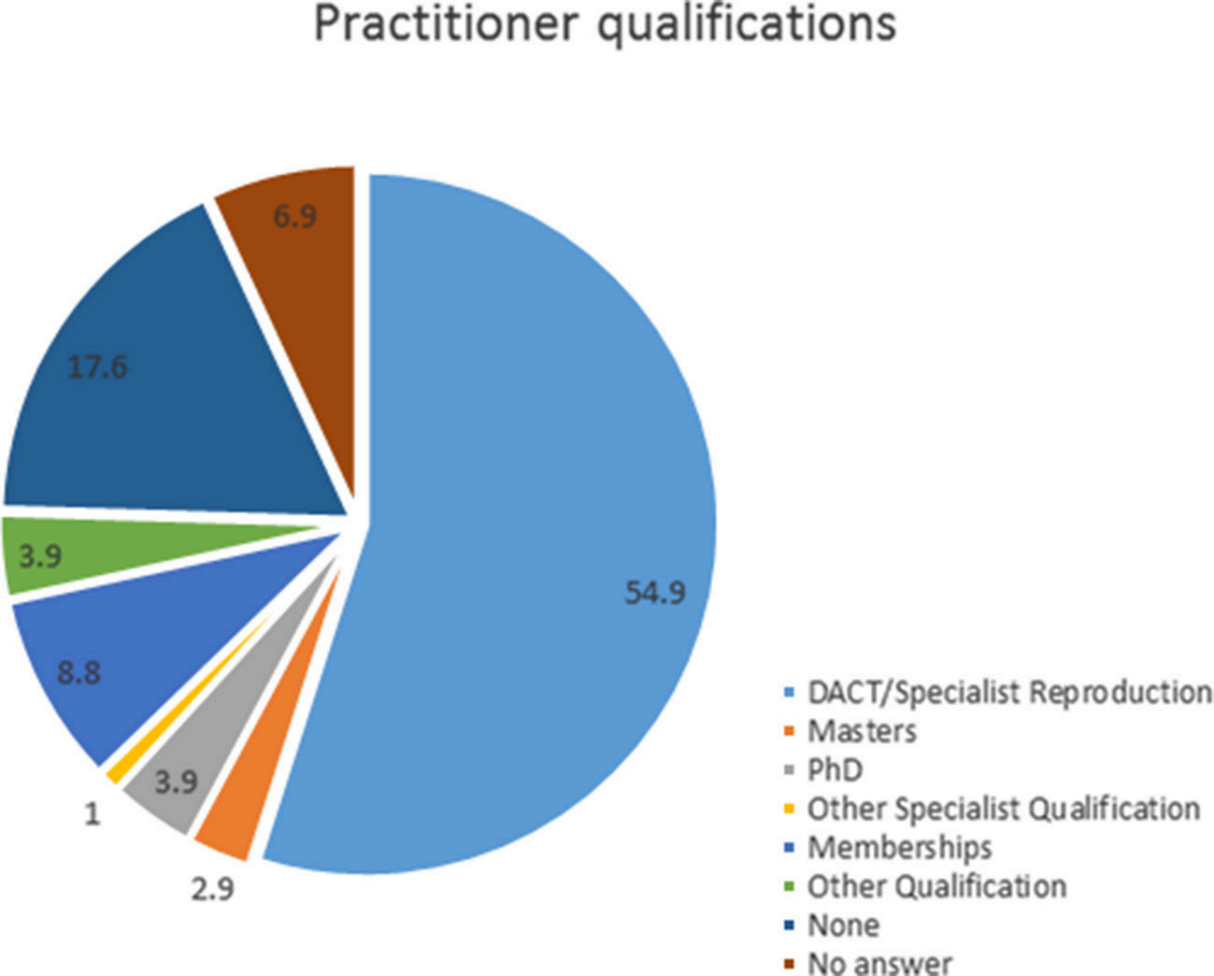
Percentage of respondents based on their highest obtained additional qualification post-veterinary degree (*n* = 102).

A total of 102 respondents partially or fully completed the survey. All complete answers were taken into account even if the survey was not filled out entirely. The number of respondents for each question are given in the results. The majority of respondents practiced in Australia (45%) and the USA (41%). While the experience of practitioners varied, nearly 58% of respondents had >20 years experience in veterinary practice. A majority of respondents had multiple qualifications. In this study, only the highest qualification was analyzed (Figure 1). Just over half of the respondents were reproductive specialists (55/102). Ninety-nine percent of respondents completed the questionnaire online.

### Diagnosis

The majority of surveyed practitioners thought that RFMs are an emergency with 66% (55/83) stating that they consider membranes left for >4 h retained. The rest of the respondents considered up to 12 h normal.

Multivariable analysis including number of years in practice, country of practice, and type of practitioner was performed using a linear regression model. Only practitioner type had a significant influence on the time until RFM was diagnosed. Specialists consider membranes to be retained after a significantly shorter period than non-specialists (*P* = 0.04) (Table 1).

**Table 1:** Univariate analyses of factors influencing the number of hours after parturition until fetal membranes are considered retained by practitioners.

	**Hours to RFM^a^**	***P*-values**
**NUMBER OF YEARS IN PRACTICE**		
< 5	3.0	Reference
6–10	4.3	0.2
11–20	4.4	0.1
>20	4.1	0.2
**COUNTRY OF PRACTICE**		
Australia	4.3	0.38
USA	3.9	
**TYPE OF PRACTITIONER**		
Specialist	3.8	0.04
Non-specialist	5.0	

a *Number of hours post-parturition when membranes are considered retained*.

**Figure 2:**
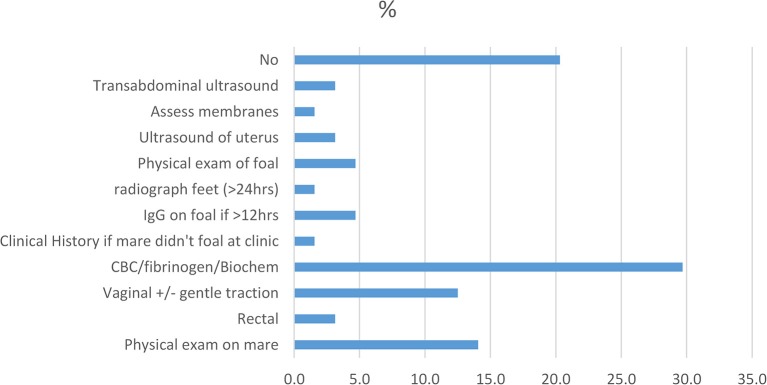
Diagnostics performed by practitioners in mares with RFM (*n* = 64).

Diagnostic tests to confirm RFM are not seen as necessary by most practitioners. However, a large proportion of practitioners performs additional tests to gage a mare's overall health status if clinical observation or history (if mare foaled elsewhere) suggest she is unwell (Figure 2). Blood tests [complete blood count (CBC), biochemistry, and fibrinogen] were the most popular additional diagnostic tool used by nearly 30% (19/64) of the respondents. A physical exam of the horse (14%, 9/64) and vaginal palpation (12.5%, 8/64) were other commonly performed tests. Some practitioners considered a physical exam on the foal as well as an IgG-test to be important if the membranes were retained for a “particularly long” period. No explanation was given for this. Interestingly, 20% (13/64) of the respondents did not believe any diagnostics needed to be performed. However, no clarification was provided as to whether clinical history and/or clinical observation were used as initial tools for the diagnosis.

### Treatments

Oxytocin is considered the most important initial treatment by most respondents. Eighty-nine percent of respondents administer varying doses of oxytocin to mares with RFM. The most commonly reported doses were 20 international units (IU) (47%, 37/79) and 10 IU (35%, 28/79) administered as IM (49%, 34/70) or IV injections (34%, 24/70). The most commonly reported frequency of oxytocin treatment ranged from 30 min to 2 h intervals as practiced by 63% (39/62) of respondents. Number of years of experience and practitioner type had no influence on initial dose of oxytocin. However, practitioners from the USA reported to use significantly lower doses than those in Australia (14 vs. 21 IU, respectively). Only three respondents administer calcium alongside oxytocin.

Traction and manual removal are attempted by 30% (27/90) of the respondents. Of those, over half reported to apply mild traction only while 37% attempt complete removal.

Flushing of the uterus is performed by 25% (22/88) of respondents with 22% (*n* = 5) of those using the Burn's technique. Large volume lavage seems to be the most common technique used by more than half of all the respondents.

Prophylactic treatments to prevent systemic diseases such as laminitis are considered important by the majority of surveyed practitioners. Antibiotics are used by 42% (37/88) of respondents with a variety of types, frequencies, and routes reported. All used antibiotics are broad spectrum and some are used in combination to increase the spectrum of susceptible species. By far the most commonly used antibiotic therapy in RFM is the combination of penicillin IM and gentamicin IV (54%, 20/37) at doses of 22,000 IU/kg BID and 6.6 mg/kg SID, respectively. Trimethoprim-sulfamethoxazole is another commonly used antibiotic preferred by 30% (11/37) of practitioners who used antibiotics in the treatment of RFM. Four respondents use uterine pessaries containing oxytocin. NSAIDs are used by 35% (31/89) of respondents with the vast majority preferring flunixin. The most commonly reported dosing regimen was 1.1 mg/kg BID IV.

In cases in which the initial treatment fails, consecutive treatment choices vary quite significantly. Only 49% (42/86) of the respondents administer oxytocin again in these cases. A much wider range of dosing regimens are used at this time compared to initial treatments. A lot more respondents use high doses of oxytocin often administered in a bag of fluids. Of the respondents that administer oxytocin again, 31% (13/42) administer doses up to and in excess of 80 IU. The most common route of administration at this point in the treatment was in a 1 l bag of lactated ringers' solution over 45 min. Doses of 20 IU were reported by 31% (13/42) of respondents while 24% (10/42) reportedly used 10 IU. The reported frequency of administration is less frequent. Traction and manual removal are attempted by a similar number of respondents as initially at 31% (27/87). Fifty-two percent of respondents attempt complete removal at this time. A much larger proportion of respondents (60%, 52/87) state the importance of flushing the uterus as part of their treatment. Nearly 31% (16/52) of these elect the Burns technique at this time in the treatment. Large volume lavage is still the preferred option for uterine flushes. Antibiotics and NSAIDs as prophylactic treatments are used a lot more commonly when initial treatment has failed. Broad spectrum antibiotics are used by 62% (53/85) of respondents with similar types and dosing regimens as reported initially. Over half of respondents use anti-inflammatories with most still preferring flunixin.

The use of the Burns technique was influenced by the number of years in practice with a significantly larger proportion of recent graduates reporting its use. In contrast, country of practice and practitioner type had no influence (Table 2).

**Table 2:** Percentage of practitioners using Burns technique for uterine flushes and attempting manual removal of fetal membranes based on univariate analysis of the factors number of years in practice, country of practice, and type of practitioner.

**Analyzed factor**	**Using Burns technique (%)**	***P*-values**	**Attempting manual removal (%)**	***P*-values**
**NUMBER OF YEARS IN PRACTICE**				
< 5	33.0	Reference	66.7	Reference
6–10	0	0.9	36.4	0.2
11–20	5.6	0.1	55.6	0.6
>20	8.8	0.1	38.2	0.2
**COUNTRY OF PRACTICE**				
Australia	9.0	0.91	63.4	0.003
USA	8.3		27.7	
**TYPE OF PRACTITIONER**				
Specialist	8.1	0.9	34.7	0.05
Non-specialist	7.1		64.3	

Manual removal/traction was used by significantly more practitioners in Australia than in the US (Table 2).

Almost half of the respondents (49%, 41/84) consider the passing of the membranes to be the end of the treatment for RFM. Another measure of successful completion of the treatment is clear lavage fluid returning from the uterus or that the mare looks recovered. Some practitioners treat for a set number of days after expulsion of the membranes, most commonly 1–2 days (11%, 9/82). Some practitioners consider treatment for up to 5 days after expulsion of the membranes.

### Complications

Three quarters of respondents stated that RFM can lead to significant medical complications in the mare. Laminitis (76%, 60/79), metritis (47%, 37/79), and toxemia/septicemia (37%, 29/79) are considered the most common complications. Twenty-seven percent of respondents reported these complications to occur most commonly within 1–2 days post-partum, while 16% (12/75) thought they were most common between 3 and 5 days post-partum. Twelve percent (9/75) reported complications were mainly seen if membranes were retained for >24 h. A majority of respondents (71%) believed that it's the failure to treat early that has led to these complications.

### Pre-dispositions and side effects

Fifty-three percent (43/81) of respondents nominated draft breeds to be most commonly affected by RFM with Friesians being considered the most commonly affected breed. A third of respondents think there are no breed pre-dispositions.

When asked whether mares with RFM had an increased risk of RFM at the next foaling, a majority of respondents (62%, 52/84) said no. When queried about the overall effects of RFM on fertility, 35% (29/84) of respondents thought there were no effects while most of the others believed otherwise. Of these, the perceived effect of RFM on fertility ranged from just affecting foal heat to affecting fertility throughout the entire breeding season.

Conditions around parturition that were believed to pre-dispose a mare to RFM were gaged with an open-ended question. A third of respondents didn't believe any conditions increased the likelihood to develop RFM. A large range of conditions were mentioned by other practitioners as pre-disposing factors. The most commonly mentioned factor was dystocia (38%, 31/82). Other conditions mentioned in this context include abortion/still birth, assisted delivery and inductions/pre-mature birth. Body condition (poor or excessive) was also a significant factor with 12% of practitioners (10/83) believing this to contribute to RFM.

### Techniques abandoned

More than half of respondents (54% *n* = 43) said that they have not abandoned any previously used techniques. Those that did stop using a technique, most commonly mentioned the Burns technique, repeated large oxytocin boluses, and manual traction.

## Discussion

Retained fetal membranes in mares are an emergency, but the treatment varies significantly (4). The changing recommendations in the literature lead to a great variation of used techniques and understanding in practitioners (16). This survey reports on the main aspects of the condition with a particular focus on treatment.

### Respondent characteristics

With a good mixture of practitioners from Australia, the US, and other countries this survey provides a broad overview of the understanding of RFM in equine practice. It is important to note that a majority of respondents are reproduction specialists, so the modes of treatments can't necessarily be extrapolated into general equine practice due to the lack of equipment and other services usually available to specialists. Specialists may also keep more up to date with the changing literature. Most of the respondents are experienced, with the majority practicing for >20 years.

### Diagnosis

The number of hours after which fetal membranes are considered retained is not well-defined. Most literature and textbooks suggest 3 h as the limit for normal expulsion of the membranes (3, 7, 22). Four to six hours post-partum have also been proposed as still being within the physiological range (7, 22). Personal communications suggest that some specialist theriogeniologists already consider membranes retained if not expulsed by 1 h post-partum, which is supported by the findings of a recent study that reported fetal membrane release within 1 h in two-thirds of mares (5).

The data collected in this survey suggest that practitioners with < 5 years of experience are most in line with the current literature with membranes suggested to be retained after an average of 3 h post-partum. The majority of practitioners with > 5 years experience consider membranes to be retained after >4 h. The earlier intervention by less experienced veterinarians may be due to a combination of less confidence of waiting it out and a greater knowledge of the current treatment suggestions, as they had been taught relatively recently. Interestingly, specialists consider membranes retained after an average of 3.8 h while non-specialists only consider them retained after an average of 5 h. This may be due to time restrictions of general practitioners, who cannot afford to treat every potential emergency as intensively. Another factor leading to earlier intervention by specialists could be the greater exposure to cases with complications post-RFM as part of a referral practice in which more specialists tend to be employed.

Diagnostic testing should always be performed if finances allow it, particularly if the mare shows systemic signs of illness (22). The most commonly reported additional diagnostic tests were blood tests including CBCs, biochemistry, and fibrinogen. Blood testing is used to estimate the systemic parameters of the mare's health to judge if any of the complications associated with RFM have occurred (12). It is surprising that there wasn't a bigger percentage of practitioners using blood tests as part of their diagnostic work up, particularly of clinically unwell mares. This might be partially due to a misunderstanding of the question. A physical exam including a vaginal exam were the next most commonly reported diagnostic tools. Again, it is somewhat surprising that this tool was not utilized by more practitioners. A physical exam of the foal and testing for IgG levels were also reported by a number of respondents. This shows the implications of the mare's health on the foal's well-being, particularly its ability to nurse effectively.

### Treatment

Treatment options were the main focus of this report. The literature considers oxytocin, uterine lavage, and antibiotics to be the triad of treatments for all RFM cases (3, 10, 12, 22). Oxytocin has previously been described as the most important initial treatment for RFM (3, 7, 22). This is clearly reflected in this survey in which 89% of respondents report to administer it to mares with RFM. Nearly half of the respondents stated the use of a dose of 20 IU IM, which is considered high dose treatment. High doses of oxytocin have to be administered carefully due to resulting strong uterine contractions potentially causing colic signs (7). More recent studies recommend very low doses of between 2 and 10 IU IM (7). Higher doses have been shown to be ineffective at expelling fetal membranes. It is hypothesized that they may cause uterine spasms instead of contractions (7). The high proportion of respondents that use 20 IU of oxytocin may be a reflection of time constraints by practitioners pressured by owners wanting RFM resolved without multiple visits from the veterinarian. Interestingly, Australian practitioners used a higher dose than those in the USA (21 vs. 14 IU, *P*-value 0.05). A bias may have been introduced due to the fact that a greater proportion of the American respondents were specialists. A large percentage of respondents also administers oxytocin frequently at 30 min to 2 h intervals. RFM cases are emergencies and there are no contraindications for using oxytocin this frequently in the literature (23). The administration route that was most popular was IM, which has been suggested to be more effective than IV injections, although IV administration leads to faster action (24). Studies also recommend the use of calcium alongside oxytocin to improve uterine contractility (7), but only three respondents indicated applying this.

Only a small proportion of respondents considers manual removal and traction, which is in line with current recommendations (4, 7, 22). Most recent studies warn against manual removal due to the high risk of hemorrhage and some also advise against gentle traction. However, other papers justify manual removal in the case of general practitioners when only a few visits to a farm are possible due to cost constraints (22). Personal communication with three veterinarians at separate equine practices confirmed that general practice veterinarians strongly prefer manual removal or firm traction in cases where owners are financially constrained. The data shows that Australian practitioners and non-specialists are more likely to attempt manual removal. Again, this is likely due to the nature of general practice where time and money constraints impact treatment. The number of years' experience did not have an effect on willingness for manual removal, which is surprising. It was expected that younger veterinarians would be less willing to remove membranes manually due to current teaching practices in veterinary schools.

Only a quarter of the respondents performs uterine lavage in RFM cases, which is in contrast to recommendations in the literature advocating uterine lavage as a way to remove inflammatory materials and dilute any toxins preventing the build-up of bacteria and endotoxins (16). Large volume lavage is the preferred option amongst practitioners that do use lavages which is in line with the current literature. A majority of respondents reported the use of sterile isotonic solutions with minimal antiseptics as has been recommended (16). The use of the Burns technique has significantly dropped in popularity due to a widely held belief that it is not as effective as normal uterine lavage. No studies have yet investigated these claims, but standard uterine lavage has been recommended (7, 16). Interestingly, the data shows that the Burns technique is most popular amongst those practitioners with less than five years of experience.

Prophylactic treatments are highly recommended by the literature with broad spectrum antibiotics and NSAIDs being the most commonly recommended drugs (3, 7, 22). They aim at preventing excessive bacterial growth and controlling endotoxin release into the bloodstream. Treatments reported in this survey are in line with these recommendations with penicillin, gentamicin, and flunixin being administered at standard doses. Trimethoprim sulfamethoxazole is another commonly used antibiotic with the benefit of being administered orally. Few practitioners included ice boots in their treatment protocol as a method for preventing laminitis. This may be due to the difficulty of using ice boots outside of clinics. Locally acting uterine pessaries are only used by a handful of respondent likely because the primary concerns in mares with RFM are the systemic effects (25).

When initial treatment fails, the majority of respondents are not willing to administer oxytocin again, perhaps due to fear about side effects and the diminished receptor receptivity several days post-partum (7). Those that administer oxytocin at this later stage prefer to use higher doses given in a bag of fluids. A larger proportion of respondents attempts complete manual removal at this stage reasoning that complications become more likely the longer the membranes are in the uterus. Flushing of the uterus in general and the Burns technique in particular become more prevalent when initial treatment has failed, likely to mitigate the increased risk of endotoxaemia. Systemic antibiotics and NSAIDs are commonly added at this stage of the treatment.

While the majority of respondents stated treating RFM mares until the membranes have passed, the literature suggests that antibiotic treatments and uterine lavage should ideally continue for an additional 1–2 weeks thereafter (7).

Since the survey has been undertaken, umbilical vessel water infusion has been reported and it will be seen if this technique holds promise in the future (26). Its use has not been mentioned by survey participants.

### Complications

Prevention of medical complications is the main concern in RFM treatment. Common complications were reported as laminitis, metritis, toxemia/septicemia in this survey, which is in agreement with the literature (6, 7, 10, 27). Most respondents indicated that complications occur most commonly in the initial days after parturition. Not surprisingly, most respondents reported that they regard the lack of early intervention/treatment as the most common cause of these complications.

### Pre-dispositions and side effects

The literature states that draft breeds such as Friesians are pre-disposed to RFM (6, 7) with which most survey respondents agree.

Several studies have shown an increased risk for repeat occurrences of RFM in future foalings of mares that developed RFM once (6, 7). Interestingly, this seems to be in complete disagreement with the experience of the majority of respondents.

A number of respondents believed that dystocia pre-disposes mares to RFM which has also been described in the literature (6). Other conditions such as abortion, stillbirths, and inductions were also mentioned in the survey as potential pre-disposing factors for RFM and have been mentioned in the literature previously (4, 27).

### Techniques abandoned

The Burns technique, manual traction, and oxytocin boluses were the most commonly reported abandoned techniques, which is in line with literature strongly recommending against these (3, 17).

## Conclusions

Various aspects of RFMs in the mare were investigated by conducting an international survey of veterinary practitioners. These included diagnosis, treatments, complications, pre-dispositions, side effects, and techniques abandoned. The discrepancy between respondents' practices and current recommendations in the literature in some aspects suggest that the increasing scientific knowledge needs to be communicated to veterinarians in practice more effectively. However, most important concepts are understood by practitioners and most treatment practices seem to be informed by and in line with current recommendations in the literature, such as low dose oxytocin administration between 2 and 10 IU IM (7) and treatment with broad spectrum antibiotics and NSAIDs (3, 7, 22). Uterine lavage, which has been recommended in the literature (16) is not being widely used by respondents. Controversial treatments such as the removal/traction of RFM are only being used by a small percentage of practitioners. Novel treatment techniques, such as the umbilical vessel water infusion technique may hold promise and should be evaluated in prospective studies. The vast variety of treatments undertaken may be partially attributed to the incomplete understanding of the condition's pathogenesis, which limits the ability to formulate evidence-based treatment recommendations.

## Ethics statement

This study was carried out in accordance with the recommendations of Minimal risk, Human Ethics Advisory Group with written informed consent from all subjects. All subjects gave written informed consent in accordance with the Declaration of Helsinki. The protocol was approved by the Human Ethics Advisory Group.

## Author contributions

NK conceived the idea and designed the survey with JM. DW and JM ran the survey and DW wrote the manuscript with guidance from NK. CM assisted in the interpretation of results and manuscript revisions. DH performed the statistical analysis and provided critical feedback throughout the study.

### Conflict of interest statement

The authors declare that the research was conducted in the absence of any commercial or financial relationships that could be construed as a potential conflict of interest.

## References

[1] Frazer GS Post partum complications in the mare. Part 2: fetal membrane retention and conditions of the gastrointestinal tract, bladder and vagina. Equine Vet Educ. (2003) 15:91–100. 10.1111/j.2042-3292.2003.tb00223.x

[2] Wright J Parturition in the mare: report based on observations made in nine normal cases. J Comp Pathol Ther. (1943) 53:212–9. 10.1016/S0368-1742(43)80020-5

[3] Threlfall WR Retained fetal membranes. In: Current Therapy in Large Animal Theriogenology. 2nd ed St. Louis, MO: Elsevier (2007), p. 107–13. 10.1016/B978-072169323-1.50015-5

[4] Threlfall WR Retained fetal membranes. In: McKinnon AO Squires EL Vaala WE Varner DD editors. Equine Reproduction. 2nd ed West Sussex: John Wiley & Sons (2011), p. 2520–9.

[5] Rosales C Krekeler N Tennent-Brown B Stevenson MA Hanlon D. Periparturient characteristics of mares and their foals on a New Zealand Thoroughbred stud farm. N Z Vet J. (2017) 65:24–29. 10.1080/00480169.2016.1244021 27705540

[6] Sevinga M Barkema H Hesselink J Retained placenta in Friesian mares: incidence, risk factors, therapy, and consequences. Pferdeheilkunde (2001) 17:619–22. 10.21836/PEM20010616

[7] Canisso IF Rodriguez JS Sanz MG Da Silva MAC A clinical approach to the diagnosis and treatment of retained fetal membranes with an emphasis placed on the critically ill mare. J Equine Vet Sci. (2013) 33:570–9. 10.1016/j.jevs.2012.08.006

[8] Rapacz-Leonard A Kankofer M Leonard M Wawrzykowski J Dabrowska M Raś A. Differences in extracellular matrix remodeling in the placenta of mares that retain fetal membranes and mares that deliver fetal membranes physiologically. Placenta (2015) 36:1167–77. 10.1016/j.placenta.2015.07.126 26297153

[9] Rapacz-Leonard A Raś A Całka J Janowski T. Expression of oxytocin receptors is greatly reduced in the placenta of heavy mares with retained fetal membranes due to secondary uterine atony. Equine Vet J. (2015) 47:623–6. 10.1111/evj.12426 25640716

[10] Provencher R Threlfall WR Murdick PW Wearly WK. Retained fetal membranes in the mare: a retrospective study. Can Vet. J. (1988) 29:903. 17423164 PMC1680927

[11] Sevinga M Vrijenhoek T Hesselink J Barkema H Groen A. Effect of inbreeding on the incidence of retained placenta in Friesian horses. J Anim Sci. (2004) 82:982–6. 10.2527/2004.824982x 15080317

[12] Leblanc MM Common peripartum problems in the mare. J Equine Vet Sci. (2008) 28:709–15. 10.1016/j.jevs.2008.10.007

[13] Asbury A The reproductive system. Equine Med Surg. (1982) 2:1305–67.

[14] Perkins NR. Equine reproductive pharmacology. Vet Clin North Am Equine Pract. (1999) 15:687–704. 10.1016/S0749-0739(17)30139-6 10589474

[15] Ros BD Willsallen C Norman ST The renaissance of oxytetracycline – a treatment for equine endometritis? In: Australian College of Veterinary Scientist Annual Conference. Gold Coast, QLD (2010), p. 70.

[16] Brinsko SP How to perform uterine lavage: indications and practical techniques. In: Proceedings of the Annual Convention of the American Association of Equine Practitioners. Lexington, KY (2001) p. 407–11.

[17] Cuervo-Arango J Newcombe JR The effect of manual removal of placenta immediately after foaling on subsequent fertility parameters in the mare. J Equine Vet Sci. (2009) 29:771–4. 10.1016/j.jevs.2009.10.004

[18] Burns S Judge N Martin J Adams L Management of retained placenta in mares. In: Proceedings of the Annual Convention of the American Association of Equine Practitioners. Lexington, KY (1978).

[19] Sager F Management and medical treatment of uterine disease. J Am Vet Med Assoc. (1968) 153:1567–9.

[20] White T Retained placenta. Wod Vet Pract. (1980) 61:87–8.

[21] Sevinga M Hesselink J Barkema H. Reproductive performance of Friesian mares after retained placenta and manual removal of the placenta. Theriogenology (2002) 57:923–30. 10.1016/S0093-691X(01)00691-4 11991394

[22] Samper J Plough T How to deal with dystocia and retained placenta in the field. In: Proceedings of the Annual Convention of the American Association of Equine Practitioners. Lexington, KY (2012), p. 359–61.

[23] Madill S Troedsson M Santschi E Malone E Dose-response effect of intramuscular oxytocin treatment on myometrial contraction of reproductively normal mares during estrus. Theriogenology (2002) 58:479–81. 10.1016/S0093-691X(02)00880-4

[24] Di Ianni F Parmigiani E Pelizzone I Bresciani C Gnudi G Volta A Comparison between intramuscular and intravenous administration of oxytocin in captive-bred red-eared sliders (*Trachemys scripta* elegans) with nonobstructive egg retention. J Exot Pet Med. (2014) 23:79–84. 10.1053/j.jepm.2013.11.009

[25] Dascanio J How and when to treat endometritis with systemic or local antibiotics. In: Proceedings of the Annual Convention of the American Association of Equine Practitioners (2011), p. 24–31.

[26] Meijer M Macpherson ML Dijkman R How to use umbilical vessel water infusion to treat retained fetal membranes in mares. In: Proceedings of the Annual Convention of the American Association of Equine Practitioners. Lexington, KY (2015), p. 478–84.

[27] Vandeplassche M Spincemaille J Bouters R. Aetiology, pathogenesis and treatment of retained placenta in the mare. Equine Vet J. (1971) 3:144–7. 10.1111/j.2042-3306.1971.tb04459.x 4949804

