# Supplementary material for: Treatment of Retained Fetal Membranes in the Mare—A Practitioner Survey
Source: Front Vet Sci. 2018 Jun 19;5:128. doi: 10.3389/fvets.2018.00128 (PMC6018472; doi:10.3389/fvets.2018.00128)
Supplement: Supplementary file 1 [file Data_Sheet_1.docx]

**Supplementary data:**

**Survey questions**

1. How many years have you been in practice?

2. Which country do you mainly practice in?

3. From which country did you graduate veterinary school?

4. Do you have any post graduate qualifications?

5. How many hours after foaling do you consider membranes to be 'retained' beyond normal?

6. Do you perform any ancillary diagnostic tests when treating for retained fetal membranes?

7. When do you initiate treatment?

8. What is your initial treatment? (please include drug names, doses, routes and frequency of administration of any drugs)

9. If your initial treatment is unsuccessful, what is your next treatment? (please include drug names, doses, routes and frequency of administration of any drugs)

10. How long do you continue to treat for retained fetal membranes?

11. In your experience, which complications do you see with retained fetal membranes?

12. When do you most commonly notice these?

13. When was the treatment initiated for retained fetal membranes in those mares?

14. Which treatments were used?

15. In your experience, is there an increased incidence of retained fetal membranes in any particular breed of mare that you see?

16. In your experience, is there an increased incidence of retained fetal membranes in mares that have been previously treated for this condition? (If so, what treatments were previously initiate

17. In your experience, do retained fetal membranes affect fertility?

18. In your experience, are there certain conditions around the time of parturition that predispose a mare to retain her fetal membranes?
